# A Novel Ruthenium-Decorating Polyoxomolybdate Cs_3_Na_6_H[Mo^VI^_14_Ru^IV^_2_O_50_(OH)_2_]·24H_2_O: An Active Heterogeneous Oxidation Catalyst for Alcohols

**DOI:** 10.3390/ma11020178

**Published:** 2018-01-23

**Authors:** Rong Wan, Qiaofei Xu, Mengdan Han, Pengtao Ma, Chao Zhang, Jingyang Niu, Jingping Wang

**Affiliations:** Henan Key Laboratory of Polyoxometalate Chemistry, Institute of Molecule and Crystal Engineering, College of Chemistry and Chemical Engineering, Henan University, Kaifeng 475004, Henan, China; wanrong1992@163.com (R.W.); xqf199408@163.com (Q.X.); m15993371075@163.com (M.H.); mpt@henu.edu.cn (P.M.); super7cc@gmail.com (C.Z.)

**Keywords:** ruthenium, polyoxomolybdate, catalyst, alcohols

## Abstract

The first example of wholly inorganic ruthenium-containing polyoxomolybdate Cs_3_Na_6_H[Mo^VI^_14_Ru^IV^_2_O_50_(OH)_2_]·24H_2_O (**1**) was isolated and systematically characterized by element analysis, infrared spectroscopy (IR), thermogravimetric analyses (TGA), X-ray photoelectron spectroscopy (XPS), energy dispersive X-ray spectroscopy (EDX) and single-crystal X-ray diffraction. Compound **1** is composed of an unprecedented {Mo_14_}-type isopolymolybdate with a di-ruthenium core precisely encapsulated in its center, exhibiting a three-tiered ladder-like structure. The title compound can act as an efficient heterogeneous catalyst in the transformation of 1-phenylethanol to acetophenone. This catalyst is also capable of being recycled and reused for at least ten cycles with its activity being retained under the optimal conditions.

## 1. Introduction

Noble metals containing polyoxometalates (POMs), in particular ruthenium (Ru)-incorporating structures [1], have been widely implemented in homo-/heterogeneous oxidation catalysis of both organic [2,3,4,5,6,7,8,9,10,11,12,13] and water-splitting reactions [14,15,16,17,18], and have therefore been attracting ever-increasing attention in recent decades. Since the discovery of the first Ru-containing POM [SiW_11_O_39_RuL]^n−^ in 1989 [2], this domain has been further explored by the research groups of Neumann, Sadakane, Bonchio, Pope, Nomiya, Proust, Hill, Bond, Kortz, Bi and others [19]. Enormous efforts have been made to check the reactivity of different ruthenium precursors, mainly using RuCl_3_·nH_2_O, (NH_4_)_2_[RuCl_6_], Ru(dmso)_4_Cl_2_ and [Ru(*η*^6^-arene)Cl_2_]_2_ with Keggin-, Dawson-, and Lindqvist-type polyanions, which has resulted in plenty of novel Ru-containing POMs. Among them, the wholly inorganic Ru-containing POMs represent an important subclass, which had been studied previously but with only a few structures reported as compared to those with inorganic-organic hybrid Ru-containing POMs. Up until now, the majority of investigations in this area of research have been dominated by the preparations and characterizations of Ru-substituted or -sandwiched lacunary Keggin/Dawson-type heteropolytungstates [2,3,4,5,6,7,8,9,10,11,12,13,14,15,16,17,18,20,21,22,23,24,25,26,27,28,29,30,31,32,33,34,35,36,37,38,39,40,41,42,43], such as mono-Ru substituted polyanions [XW_11_O_39_Ru^III^(H_2_O)]^n−^ (X = Si, Ge, P) [2,3,4,5,20,21,22,23,24,25] and [P_2_W_17_O_61_Ru^III^(H_2_O)]^5−^ [26,27,28], di-Ru-sandwiched/substituted polyanions {O[Ru^IV^(X)P_2_W_17_O_61_]_2_}^16−^ (X = OH, Cl) [27], {[(XZnRu_2_^III^(OH)(H_2_O)](ZnW_9_O_34_)_2_}^n−^ (X = W, Zn) [29,30,31,32,33,34], [{*a*-SiW_11_O_39_Ru^III^}_2_O]^12−^ [21,23], [{PW_11_O_39_}_2_{(HO)Ru^IV^}_2_O}]^10–^ [35], and [XW_10_O_38_{Ru^IV^N}_2_]^6−^ (X = Si, Ge) [36], tri-Ru-substituted polyanion [SiW_9_O_34_{Ru_3_O_3_(H_2_O)Cl_2_}]^7−^ [41], tetra-Ru-sandwiched/substituted polyanions [{Ru^IV^_4_O_4_(OH)_2_(H_2_O)_4_}(*γ*-SiW_10_O_36_)_2_]^10−^ [37,38], [Ru^IV^_4_Cl_4_O_2_(*μ*-OH)_4_(*γ*-SiW_10_O_36_)_2_]^12−^ [39], [(*γ*-PW_10_O_36_)_2_Ru^IV^_4_O_5_(OH)(H_2_O)_4_]^9−^ [40], and [SiW_9_O_37_{Ru_4_(H_2_O)_3_Cl_3_}]^7−^ [42]. However, some of the above-mentioned examples were not supported by structural confirmation evidence, or were not even capable of providing a reliable molecular formula, primarily due to the difficulties of obtaining the crystal structures. For instance, there have been some controversies regarding the reproduction and precise structure of two Ru_2_-incorporated POMs with putative formulae [WZnRu_2_^III^(H_2_O)(OH)(ZnW_9_O_34_)_2_]^11−^ and {[(Zn_2_Ru_2_^III^(OH)(H_2_O)](ZnW_9_O_34_)_2_}^14−^, which were synthesized under completely identical synthetic conditions by different research groups [26,27,28]. Recently, the same doubts were also raised with regard to the polyanions [Ru_4_(H_2_O)_3_Cl_3_(SiW_9_O_37_)]^7−^ and [SiW_9_O_37_{Ru_4_(H_2_O)_3_Cl_3_}]^7−^ owing to the absence of crystal structure [41,42]. To the best of our knowledge, only very little direct crystallographic evidence of inorganic Ru-containing POMs has been reported in early-stage investigations (Table S1). Therefore, it is still a great challenge and an excellent opportunity for us to either explore or investigate their structures along with their catalytic applications. 

Herein, we report a novel inorganic Ru-containing POM Cs_3_Na_6_H[Mo^VI^_14_Ru^IV^_2_O_50_(OH)_2_]·24H_2_O (**1**), which is the first example of X-ray crystallographically characterized inorganic Ru-containing polyoxomolybdate. The catalytic study revealed that compound **1** could efficiently catalyze 1-phenylethanol to acetophenone with excellent structural stability in the presence of tert-butyl hydroperoxide (TBHP) as an oxidant in the reaction medium.

## 2. Results and Discussion

### 2.1. Structure Description

The phase purity of sample **1** was characterized by X-ray powder diffraction, as the experimental pattern was mainly consistent with the simulated one (Figure S1). The bond valence sums (BVS) calculated for polyanion **1** from the observed bond lengths mainly indicate that all the Mo and Ru exist exclusively in +VI and +IV valence states, respectively (Table S2). Simultaneously, two *μ*_3_-OH bridges (O5 and O5A; BVS: 1.19) in the cluster were also determined by the BVS calculations (Table S3). From these results, the chemical composition of polyanion **1** was defined as [Mo^VI^_14_Ru^IV^_2_O_50_(OH)_2_]^10−^ (**1a**), which was confirmed by the combined analyses of XPS and EDX (Figure S2) [41,43].

**Figure 1:**
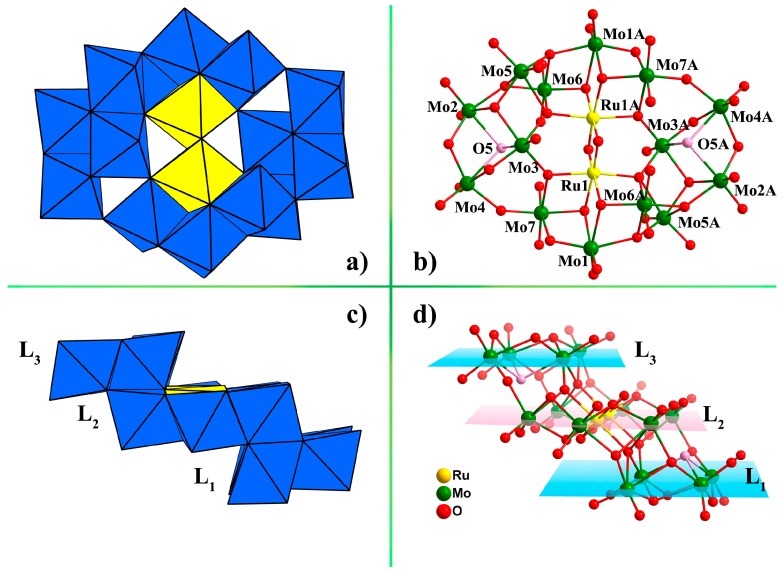
Top view of (**a**) polyhedral and (**b**) ball-and-stick representations of polyanion [Mo^VI^_14_Ru^IV^_2_O_50_(OH)_2_]^10−^ (**1a**); Side view of (**c**) polyhedral and (**d**) ball-and-stick representations of polyanion **1a**. Color code: MoO_6_ octahedral, blue; RuO_6_ octahedral, yellow. The pink balls represent monoprotonated oxygen atoms.

As illustrated in Figure 1, the di-ruthenium core was fully incorporated into the center of the {Mo_14_} cluster, which can be described as an assembly of two symmetric {Mo_7_Ru} units bridged by six oxygen atoms in a head-to-tail motif, forming a novel three-tiered ladder-like structure. The middle layer of the “stairs” ({Mo_6_Ru_2_}, denoted as L2) was defined by six coplanar Mo centers (Mo1, Mo6, Mo7, Mo1A, Mo6A, Mo7A) and two Ru centers (Ru1, Ru1A) (A = 1–X, –Y, 1‒Z). Moreover, Mo2, Mo3, Mo4 and Mo5 atoms, or their corresponding symmetrical atoms, separately gave two identical {Mo_4_} “steps”. The four MoO_6_ units in each {Mo_4_} subunit were linked together by edge sharing, resulting in a quasi-flat plane (denoted as L1 and L3). The average deviations of L1, L2 and L3 were all 0.00 Å, indicating that each of them showed excellent planarity. The dihedral angles between L1 and L3 are 0°, while the dihedral angles between L1 (or L3) and L2 were 2.36° with average spacing distance of 2.75 Å. Within each layer (Figure 1c,d), the two {Mo_4_} “steps” were added to the middle {Mo_6_Ru_2_} “step” up and down, with a certain degree of dislocation, as a result of face and vertex sharing modes, alternately, resulting in the novel ladder-shape structure. Furthermore, polyanion **1a** was linked by the Na cation cluster, leading to a 1D layer-like structure (Figure S3).

The so-called “Mo_14_Ru_2_” was first presented by Oonaka and co-workers, but was not structurally authenticated by X-ray single-crystal diffraction. Furthermore, no exact molecular formula was provided. Originally, the so-called “Mo_14_Ru_2_” was determined as Na_4_(NH_4_)[RuMo_7_O_25_]·8H_2_O; although it was subsequently identified as [Ru_2_Mo_14_O_50_]^10−^ or [Ru_2_Mo_14_O_52_]^14−^, owing to its lack of a crystal structure [44,45]. This unknown or unconfirmed structure was also commented on by Izarova, Pope and Kortz in their review of “Noble Metals in Polyoxometalates” [1]. To the best of our knowledge, compound **1** represents the first X-ray crystallographically characterized example of inorganic Ru-containing polyoxomolybdate. Moreover, the new type of {Mo_14_} unit is obviously different from that in the organic-inorganic hybrid polyoxomolybdate [Mo_14_O_38_(OAc)_6_]^2–^ recently reported by Hayashi et al. [46]. The {Mo_14_} cluster that we observed exhibits a ladder-like structure, while the hybrid polyanion [Mo_14_O_38_(OAc)_6_]^2–^ (Figure S4) can be regarded as a hexa-lacunary Dawson-type structure in which the six lacunary sites at the polar positions are supported by six acetate groups. 

### 2.2. Catalytic Performance

As already noted, POMs can be used extensively as active oxidation catalysts for organic or inorganic substrates [47,48]. We are also interested in the application of Ru-decorated POMs for alcohol oxidation, which is one a reaction of key practical importance in the chemical industry. Indeed, compound **1** exhibited highly efficient catalytic activity toward the heterogeneous oxidization of 1-phenylethanol into acetophenone (Figure S5). 

**Figure 2:**
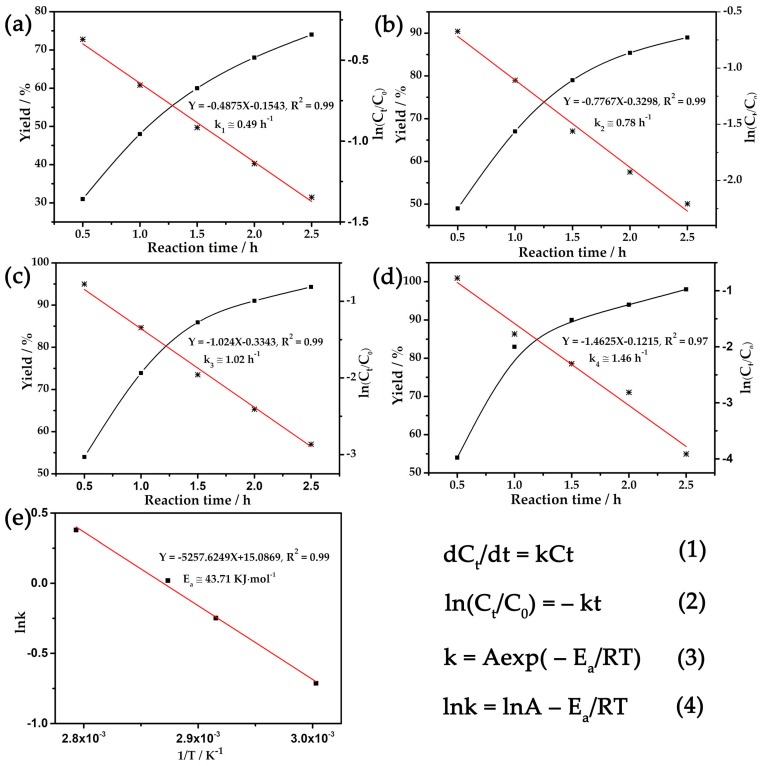
Kinetics for 1-phenylethanol oxidation reaction using catalyst **1** at (**a**) 60, (**b**) 70, (**c**) 75 and (**d**) 85 °C for 3 h, respectively. The data from each reaction temperature are fitted to a single straight line, indicating the reaction was pseudo-first-order in 1-phenylethanol. (**e**) Arrhenius plot for the oxidation of 1-phenylethanol using catalyst **1** at 60, 70, 75 and 85 °C for 3 h. C_0_ and C_t_ represent the concentrations of reactant initially and at time t, respectively.

According to Table S4, the main factors affecting the oxidation process, including the amount of catalyst and oxidizing agent TBHP, and the reaction temperature and time, were investigated in detail to explore suitable reaction conditions. Parallel experiments showed that the reaction yield improved with the increase in catalytic dosage, while declining remarkably with the decrease of the amount of oxidant, reaction temperature and time. Specifically, optimized reaction conditions have been identified, for which 0.15 mol % catalyst loading with 8 mol of TBHP in acetonitrile for 3 h at 85 °C provide an almost 100% yield from the starting substrate to the single product. Meanwhile, the chemical kinetics for the 1-phenylethanol oxidation reaction has been demonstrated as a function of time for catalyst **1** (Figure 2) [6,49,50,51,52]. Yield and ln(C_t_/C_0_) are plotted against the reaction time at 60, 70, 75, and 85 °C (Figure 2a–d), where C_0_ and C_t_ represent the concentrations of the reactant initially and at time t, respectively. The linear fit of the data reveals that the catalytic reaction follows pseudo-first-order kinetics at 60, 70, 75, and 85 °C. The absolute value of the slope of the straight line represents the reaction rate constant; namely, 0.49 h^−1^ for 60 °C, 0.78 h^−1^ for 70 °C, 1.02 h^−1^ for 75 °C and 1.46 h^−1^ for 85 °C. Furthermore, the apparent activation energy, E_a_, was determined based on the Arrhenius plot in the temperature range of 60–85 °C, and the E_a_ value was found to be 43.71 kJ mol^−^^1^ (Figure 2e). 

**Figure 3:**
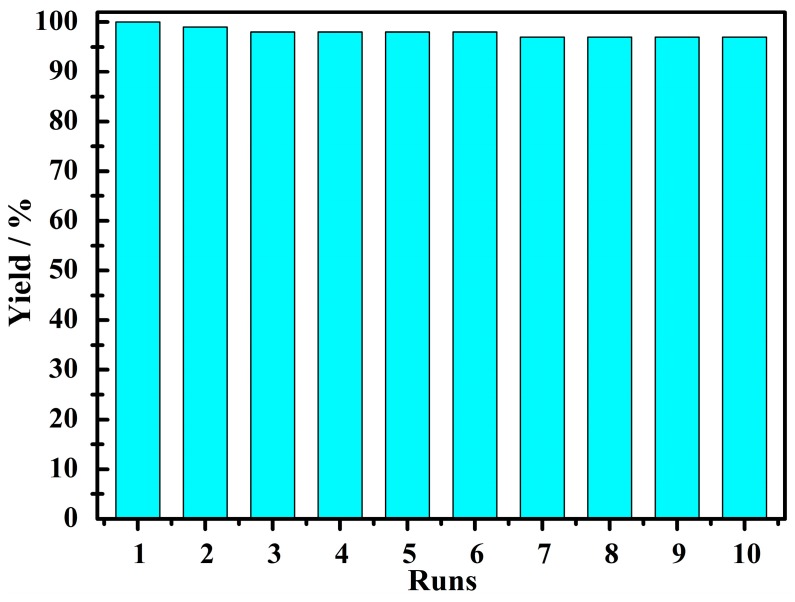
Recovery and reuse of the catalyst **1**. The GC yield (%) based on 1-phenylethanol to acetophenone under optimized reaction conditions.

**Figure 4:**
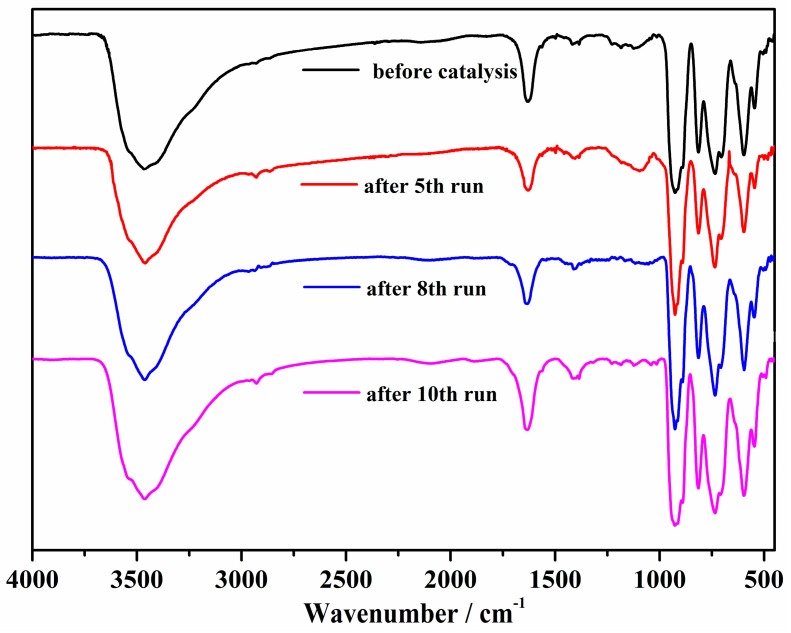
IR spectra of catalyst **1** before and after the fifth, the eighth, and the tenth run.

Afterwards, the blank experiment was carried out in the absence of compound **1** under the controlled conditions, with only a little catalytic reactivity observed in the temperature range of 60–85 °C following a reaction time of 3 h (Figure S6). We also independently investigated the catalytic properties of RuCl_3_·nH_2_O and Na_2_MoO_4_·2H_2_O for comparison (Figure S6). The control test also implied that little reaction occurred when using Na_2_MoO_4_·2H_2_O as a catalyst. Although the catalyst RuCl_3_·nH_2_O showed good catalysis for the reaction, the catalysis couldn’t be improved with the prolongation of reaction time or increase in temperature. Moreover, it is difficult to isolate and reuse, since RuCl_3_·nH_2_O is a homogeneous catalyst in the reaction medium. Therefore, catalyst **1,** on the whole, outperformed both of the synthetic materials RuCl_3_·nH_2_O and Na_2_MoO_4_·2H_2_O. The hot filtration experiment was carried out to remove the catalyst following a reaction time of 0.5 h, with the reaction then being allowed to proceed further with the filtrate under the optimal conditions. It appears that scarcely any further yield was observed in the filtrate, suggesting that this oxidation process is heterogeneous (Figure S7). More notably, a further study on the recyclability of compound **1** was carried out, which suggested that this catalyst could be recycled and reused for at least ten cycles with less than a 3% loss of activity (Figure 3). Once every round of the catalytic process was completed, the catalyst was isolated and dried, and with a characterization of the IR spectrum was performed (Figure 4), demonstrating that compound **1** possesses excellent structural stability, as no structural changes were observed after the oxidation reaction. 

Finally, in the context of the optimal conditions, we sought to evaluate the versatility of our catalyst by studying the oxidation of several substituted 1-phenylethanol with different substituents (Table 1). The results indicated that all the 1-phenylethanol with para- or meta-substituents produced their corresponding ketones derivatives in high yields (97–100%) on reaction, as well as demonstrating 100% selectivity regardless of their electronic properties (Table 1, entries 1–5). As for the ortho-substituted 1-phenylethanol with different steric and electronic properties (Table 1, entries 6–8), the reaction showed a similar moderate yield, i.e., the more sterically hindered –OH group showed less catalytic activity. This fact suggests that neither the different electronic properties nor the positions of substituents on the phenyl ring of 1-phenylethanol were key parameters for this reaction, but that the steric effect considerably hindered the catalytic activity [53]. Furthermore, compound **1** also exhibited good catalytic activity for secondary chain and cyclic aliphatic alcohols (Table 1, entries 9–12).

### 2.3. X-ray Photoelectron Spectroscopy (XPS)

**Figure 5:**
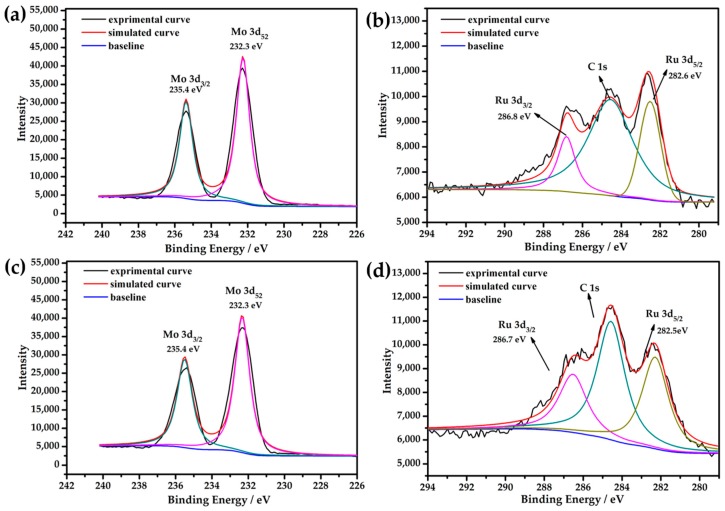
The XPS spectra for the catalyst: (**a**) Mo 3d and (**b**) Ru 3d before reaction; (**c**) Mo 3d and (**d**) Ru 3d after the tenth run of the reaction. Spectral analysis was performed using peak-fitting with Gaussian–Lorentzian peak shape and Shirley-type background subtraction, taking as reference the C 1s peak typically at 284.6 eV and the Ru 3d_5/2_, 3d_3/2_ double peak with a constant area rule: A(3d_5/2_) = A(3d_3/2_)/1.5 and center position relation χ_c_(3d_5/2_) = χ_c_ (3d_3/2_) + 4.2 eV.

The XPS spectra for Mo 3d and Ru 3d of the catalyst before and after the catalytic reaction were measured in order to check the surface oxidation state of the solid and to further verify the stability of the well-defined cluster (Figure 5). Before reaction, the XPS spectra of the Mo centers had the following characteristics: the peaks around 235.4 eV and 232.3 eV in the energy regions of Mo 3d_3/2_ and Mo 3d_5/2_ are assigned to Mo^VI^ centers represented in Figure 5a [54,55], which is consistent with the BVS results. Although the oxidation of Ru^III^ to Ru^IV^ in aqueous solution is known in POMs chemistry [24,27,38], a high-resolution Ru 3d XPS spectrum was nevertheless obtained to further characterize their oxidation states, as Ru is a highly specific element, and is known to have a wide range of oxidation states varying from –II to VIII. As shown in Figure 4b, the Ru 3d peaks in the high-resolution XPS spectra of the title compound centered on 286.8 eV and 282.6 eV, corresponding to energy regions of Ru 3d_3/2_ and Ru 3d_5/2_, which are attributed to Ru^IV^ centers [43,56,57]. This oxidation state of Ru assignment was also supported by the BVS results, which indicated a IV valence [38,58].

To investigate the stabilization of **1** for catalysis, catalyst **1** was dried in a vacuum oven following the tenth run of the reaction, and was then characterized based on its high-resolution XPS spectra. The Mo 3d and Ru 3d XPS spectra (Figure 5c,d) for the recovered catalyst after reaction were clearly almost identical to those of the fresh catalyst (Figure 5a,b), indicating that the states of the surface of the catalyst had undergone no change; in other words, catalyst **1** was stable in the selected reaction system.

### 2.4. Thermogravimetric Analysis (TGA)

**Figure 6:**
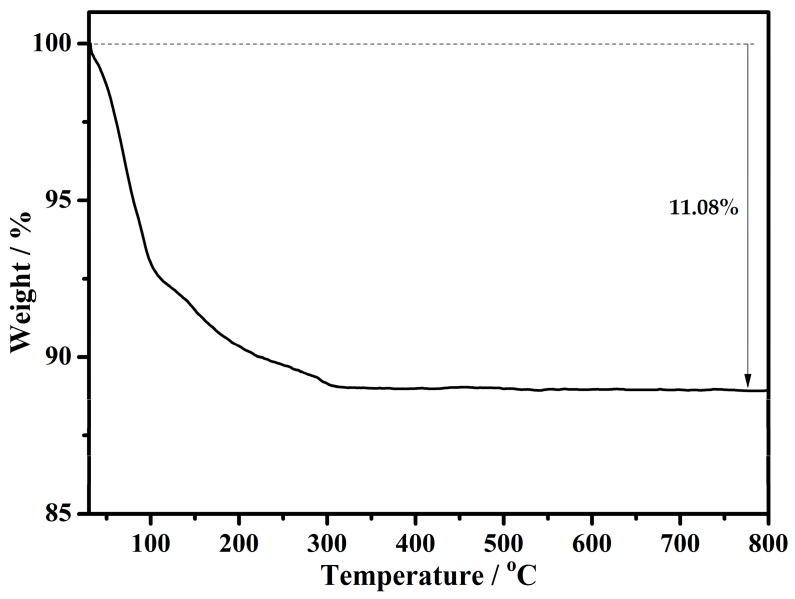
TG curve of compound **1**.

The thermal stability of the title compound was investigated on crystalline samples under a nitrogen atmosphere from 30 to 800 °C with a heating rate of 10 °C min^−1^. The TG curve indicates that compound **1** undergoes a successive one-step weight-loss process (Figure 6), and that the weight loss of 11.08% is attributable to the release of 21 water molecules. The measured weight loss (11.08%) is a bit lower than the theoretical weight loss (24 lattice water molecules, calcd 12.91%), which is probably due to the slight weathering of the title compound. 

## 3. Materials and Methods

### 3.1. Synthesis of Compound ***1***

Na_2_Mo^VI^O_4_·2H_2_O (0.968 g, 3.00 mmol), Ru^III^Cl_3_·nH_2_O (0.120 g, 0.58 mmol) was successively dissolved in 20 mL distilled water while vigorously stirring at room temperature, resulting in a dark solution. The pH value of this solution was adjusted to around 5.0 with 3 mol·L^−1^ NaOH solution. After heating in the water-bath of 80 °C for 2 h, ten drops of 1 mol·L^−1^ CsCl solution was added and stirred with about fifteen minutes at room temperature. Then, the solution was filtered and left to crystallize slowly. Black block crystals were collected after about one week. Yield: 0.34 g (47.42% based on Mo). Elemental analysis calcd (%) for 1: Na 4.12, Mo 40.08, Ru 6.03, Cs 11.90. Found: Na 3.86, Mo 40.02, Ru 5.93, Cs 11.75. IR (cm^−1^): 3468 (vs), 1622 (m), 926 (vs), 889 (s), 822 (m), 744 (s), 594 (m). The IR spectrum exhibited two kinds of characteristic bands attributed to ν (Mo = O) and ν (Mo–O–Mo) at 926 and 889‒594 cm^−1^, respectively.

### 3.2. Characterization

All reagents used were of commercial reagent grade, and were used without further purification for the preparation of the title compound. Elemental analysis of Mo, Ru, Na and Cs atoms was conducted on a Perkin Eimer Optima 2100 DV inductively coupled plasma optical emission spectrometer (Perkin-Elmer, 940 Winter Street Waltham, MA, USA). IR spectra were recorded on a Bruker VERTEX 70 IR spectrometer (Nicolet, Madison, WI, USA) in the range of 4000‒450 cm^−1^ with pressed KBr pellets. XRPD data were performed on a Bruker AXS D8 Advance diffractometer (Bruker, Karlsruhe, Germany) with Cu Kα radiation in the angular range 2θ = 5°–45° at room temperature. TG analysis was measured on a NETZSCH STA449F5/QMS403D instrument (Mettler-Toledo, Sonnenbergstrasse 74, Schwerzenbach, Switzerland) with a heating rate of 10 °C·min^−1^ in flowing nitrogen. Energy-dispersive X-ray spectroscopy (EDX) measurements were performed with a JSM-7610F scanning electron microscope (JEOL, Tokyo, Japan) using an OXFORD X-act EDX. X-ray photoelectron spectroscopy (XPS) was performed on an Axis Ultra (Kratos, Manchester, UK) X-ray photoelectron spectroscope using monochromatic Al K*α* (1486.7 eV) radiation. 

### 3.3. Crystallography

A single crystal of **1** was sealed in a tube capillary when prepared for data collection at 296(2) K. Intensity data collection was performed on a Bruker APEX-II CCD diffractometer (Bruker-AXS, Karlsruhe, Germany) with graphite-monochromated Mo K*α* radiation (λ = 0.71073 Å). Structure solution and refinement were carried out using the SHELXL-2014/7 program package (University of Göttingen, Göttingen, Germany) [59,60]. In the final refinement cycles, the Mo, Ru, Na and Cs atoms were refined anisotropically. All H atoms on water molecules were incorporated directly into the molecular formula. Crystallographic data for the structure reported in this paper have been deposited in the Cambridge Crystallographic Data Center, with a CCDC number of 1584135 for **1**. Crystal data and structure refinement parameters are listed in Table S5.

### 3.4. General Procedure for Catalysis

The typical experimental procedure for the catalytic oxidation of various secondary aromatic alcohols was carried out in a 50 mL round-bottomed tube equipped with a reflux condenser. Generally, 0.015 mmol of catalyst, 1 mmol of alcohols, 8 mmol of TBHP and 3 mL acetonitrile were charged in the reaction tube at the designated temperature with constant stirring throughout the whole reaction. At regular intervals, an aliquot of the sample solution was taken directly from the reaction mixture with a microsyringe and the liquid was analyzed by gas chromatography (GC) using dodecane as the internal standard. As for the recycling experiment, the POM catalyst was recovered by filtration when the reaction mixture was cooled to room temperature at the end of each cycle, and then washed thoroughly (at least three times) by acetonitrile, which was further dried at 70 °C in oven and reused for successive runs under identical reaction conditions.

## 4. Conclusions

In summary, a new type of pure inorganic Ru-containing POM [Mo^VI^_14_Ru^IV^_2_O_50_(OH)_2_]^10−^ was described in this work, which is the first X-ray crystallographically characterized example of an inorganic Ru-containing polyoxomolybdate. This compound is a highly efficient and recyclable catalyst for oxidizing 1-phenylethanol to acetophenone. Further research will concentrate on exploring the synthesis of novel Ru-containing POMs and expanding the application range of catalytic reaction types, which is an opportunity for us, even if it is still a challenging task now and in the near future. 

## Figures and Tables

**Table 1:** Substrate scope for aromatic secondary alcohols in the context of the optimal conditions ^a^.

Entry	Substrate	Product	Yield (%)	Entry	Substrate	Product	Yield (%)
1	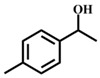	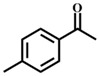	99	7	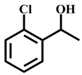	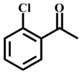	58
2	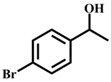	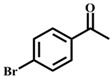	99	8	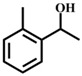	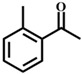	52
3	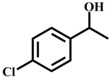	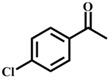	97	9	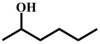	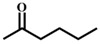	70
4	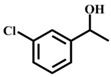	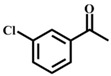	100	10	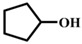	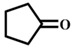	70
5	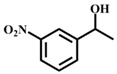	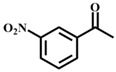	97	11	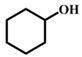	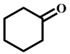	76
6	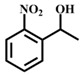	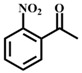	46	12	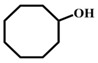	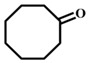	97

^a^ eaction conditions for the entries 1 to 12: Catalyst, 0.15 mol %; substrate, 1 mmol; acetonitrile, 3 mL; TPHB, 8 mmol; reaction temperature, 85 °C; reaction time, 3 h. All of the products were identified by their GC spectra.

## Supplementary Materials

The following are available online at http://www.mdpi.com/1996-1944/11/2/178/s1, Figure S1: The experimental and simulated XRD patterns of compound **1**; Figure S2: The EDX spectrum of compound **1**; Figure S3: The 1D chain-like structure of polyanion **1a**; Figure S4: The structural comparison of polyanion **1a** and polyanion [Mo_14_O_38_(OAc)_6_]^2–^; Figure S5: GC spectra of (a) standard 1-phenylethanol; (b) standard acetophenone; (c) the oxidation of 1-phenylethanol to acetophenone product; Figure S6: (a) Contrast experiments with different catalysts at different reaction times, (b) Contrast experiments with different catalysts at different reaction temperatures; Figure S7: The reaction results of the hot filtration test; Table S1: Summary of pure inorganic ruthenium-containing POMs with well-defined structures; Table S2: The bond valence sum calculations of all crystallographically unique Mo and Ru atoms on **1a**; Table S3: The bond valence sum calculations of all crystallographically unique O atoms on **1a**; Table S4: Optimization of catalytic oxidation of 1-phenylethanol using catalyst **1**. Table S5: Crystallographic data of compound **1**.
