# Supplementary material for: A Novel Ruthenium-Decorating Polyoxomolybdate Cs3Na6H[MoVI14RuIV2O50(OH)2]·24H2O: An Active Heterogeneous Oxidation Catalyst for Alcohols
Source: Materials (Basel). 2018 Jan 23;11(2):178. doi: 10.3390/ma11020178 (PMC5848875; doi:10.3390/ma11020178)
Supplement: Supplementary file 1 [file materials-11-00178-s001.pdf]

Supporting Information

A novel ruthenium-decorating polyoxomolybdate $Cs_3Na_6H[Mo^{VI}_{14}Ru^{IV}_2O_{50}(OH)_2]\cdot 24H_2O$: an active heterogeneous oxidation catalyst for alcohols

Rong Wan, Qiaofei Xu, Mengdan Han, Pengtao Ma, Chao Zhang, Jingyang Niu* and Jingping Wang*[a]

[a]Henan Key Laboratory of Polyoxometalate Chemistry, Institute of Molecular and Crystal Engineering, College of Chemistry and Chemical Engineering, Henan University, Kaifeng, Henan 475004, (P. R. China), Fax: (+86)-371-23886876, E-mail: jyniu@henu.edu.cn, jpwang@henu.edu.cn.

CONTENTS:

Figure S1. The experimental and simulated XRD patterns of compound **1**;

Figure S2. The EDX spectrum of compound **1**;

Figure S3. The 1D chain-like structure of **1a**;

Figure S4. The structural comparison of (a) polyanion **1a** and (b) polyanion $[Mo_{14}O_{38}(OAc)_6]^{2-}$;

Figure S5. GC spectra of (a) standard 1-phenylethanol; (b) standard acetophenone; (c) the oxidation of 1-phenylethanol to acetophenone product;

Figure S6. (a) Contrast experiments with different catalysts at different reaction times; (b) Contrast experiments with different catalysts at different reaction temperatures.

Figure S7. The reaction results of the hot filtration test;

Table S1 Summary of pure inorganic ruthenium-containing POMs with well-defined structures;

Table S2 The bond valence sum calculations of all crystallographically unique Mo and Ru atoms on **1a**;

Table S3 The bond valence sum calculations of all crystallographically unique O atoms on **1a**;

Table S4 Optimization of catalytic oxidation of 1-phenylethanol;

Table S5 Crystallographic data of **1**.

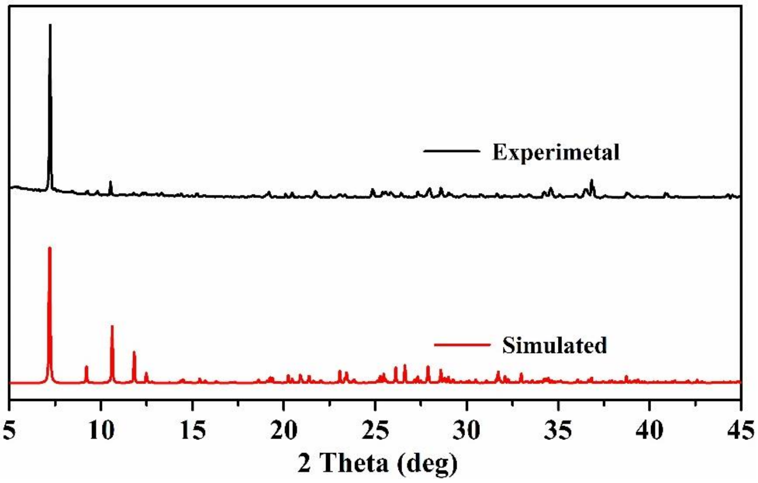

Figure S1. The experimental and simulated XRD patterns of compound **1**.

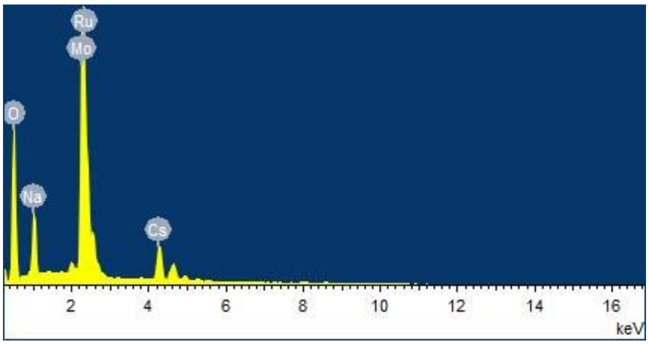

Figure S2. The EDX spectrum of compound **1.**

We used EDX to characterize the composition of compound **1**. According to the EDX spectrum, Mo and Ru were detected with approximate ratio of Mo: Ru found to be 6.4: 1 and this result has been checked using multiple samples to reduce the error in the value. However, as we know, the EDX measurements have about 8% error, which is estimated on comparison with other reported POMs. Therefore, using both the structural data and analysis results of various techniques including ICP, EDX, XPS, charge balance arguments, we are able to estimate the formula of the title compound. Using this approach we can give the formula of **1** as $Cs_3Na_6H[Mo^{VI}_{14}Ru^{IV}_2O_{50}(OH)_2]\cdot24H_2O$ (Mo : Ru = 6.6 : 1).

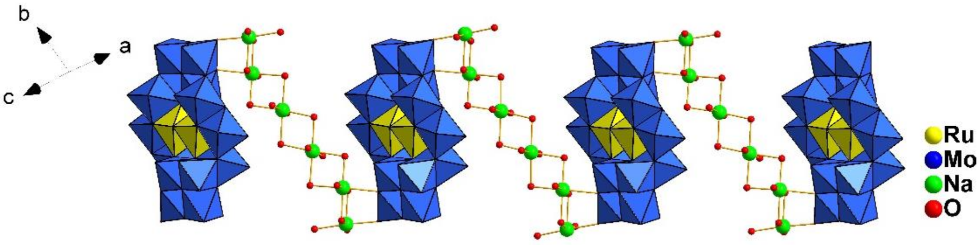

Figure S3. The 1D chain-like structure of **1a**.

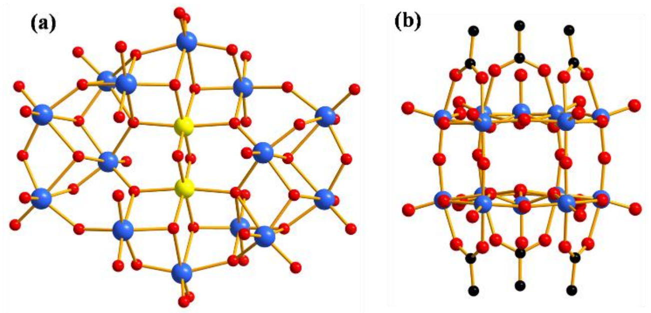

Figure S4. The structural comparison of (a) polyanion **1a** and (b) polyanion $[Mo_{14}O_{38}(OAc)_6]^{2-}$.

Gas chromatographic analysis

GC conditions for standard 1-phenylethanol, standard acetophenone and the oxidation of 1-phenylethanol to acetophenone product. Column information GsBP-5, L = 30 m, 0.25 mmID, injector temperature 300 °C, column flow rate 1.80 ml/min, column initial temperature 50 °C, temperature program 8 °C/min, detector temperature 300 °C.

According to Figure S5a and S5b, the retention of standard 1-phenylethanol, standard acetophenone in the selected condition are at 7.30 (±0.03) and 7.40 (±0.03) min, respectively. The peaks at 1.63 min in both Figure S5a and S5b are attributed to the solvent of acetonitrile. Figure S5c showed GC spectra for the oxidation of 1-phenylethanol to acetophenone product. There is a single peak of product centered at 7.43 (±0.03) min, which is almost identical to that of standard acetophenone (Figure S5b), indicating that the title compound can efficiently catalyze oxidizing 1-phenylethanol merely to acetophenone. In addition, the peaks appeared at 1.63, 1.67, 2.41, and 6.23 min are corresponding to acetonitrile, tert-butyl alcohol (the reduction product of TBHP), and TBHP, and decane (serves as a protectant for TBHP).

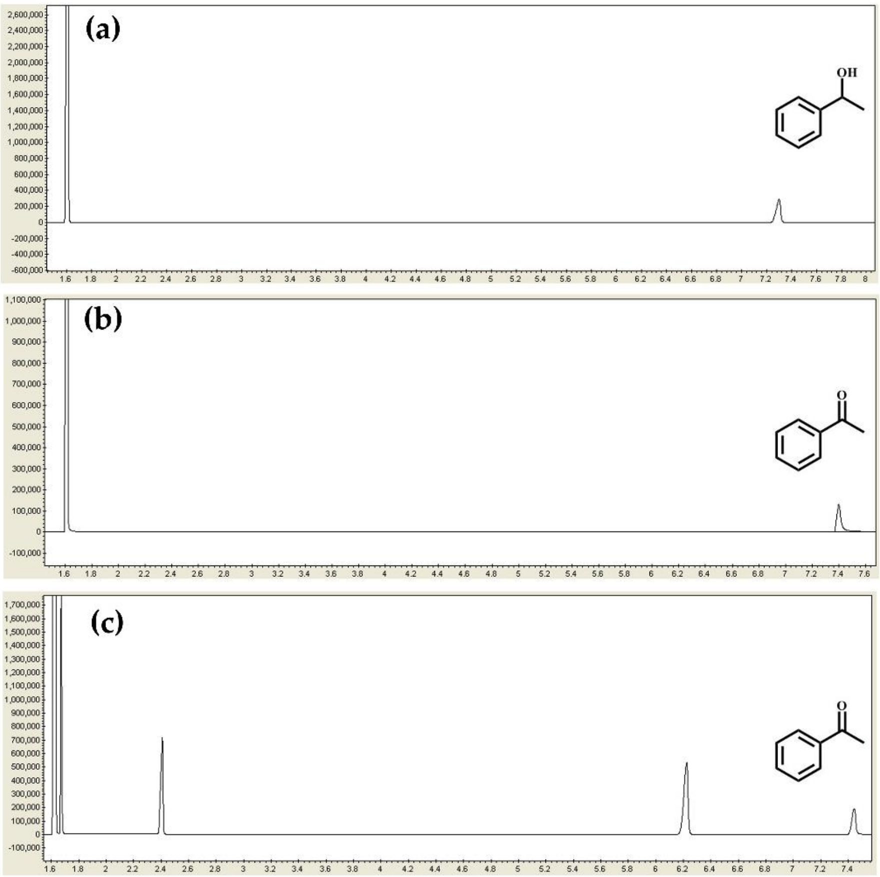

Figure S5. GC spectrum of (a) standard 1-phenylethanol; (b) standard acetophenone; (c) the oxidation of 1-phenylethanol to acetophenone product.

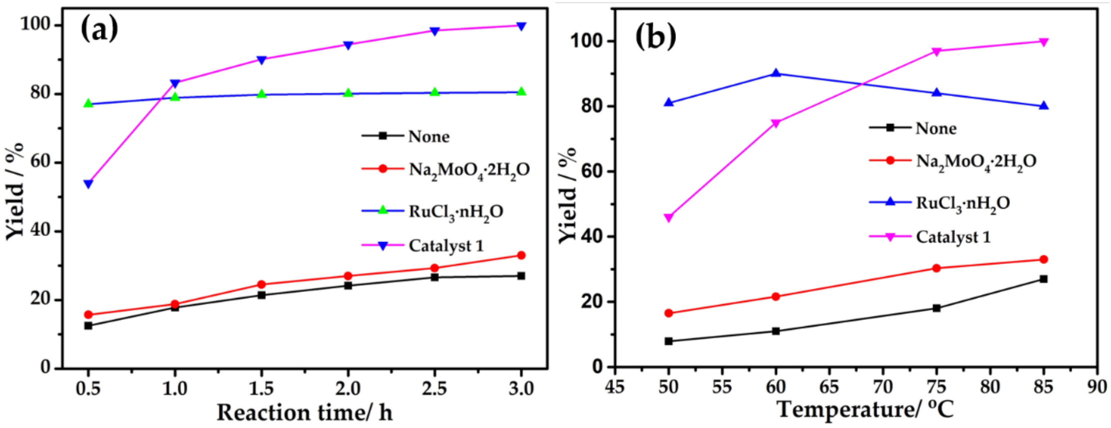

Figure S6. (a) Contrast experiments with different catalysts at different reaction times. Reaction condition: substrate (1 mmol) and acetonitrile (3 mL), temperature (85 °C); (b) Contrast experiments with different catalysts at different reaction temperatures. Reaction condition: substrate (1 mmol) and acetonitrile (3 mL), time (3 h). Yield determined by GC with dodecane as an internal standard.

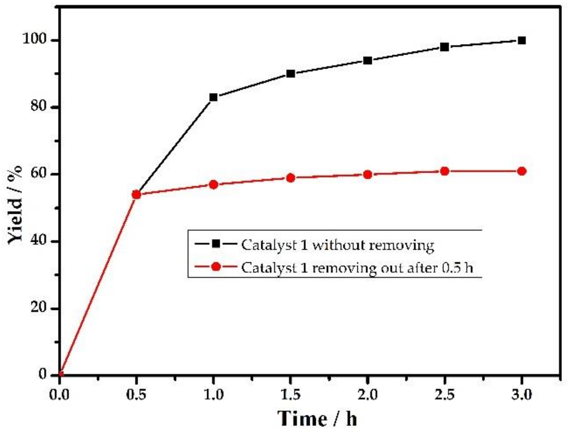

Figure S7. The reaction results of the hot filtration test.

The hot filtration test

As POM catalyst **1** is insoluble in the reaction system and can be recovered by simple centrifugation and dried, the hot filtration experiment was carried out to remove the catalyst at a reaction time of 0.5 h and the reaction was further allowed to proceed with the filtrate under the optimal conditions. It appears that scarcely no obvious conversion was observed in the filtrate, suggesting that the epoxidation process in this study is heterogeneous (Fig. S7).

Table S1. Summary of pure inorganic ruthenium-containing POMs with well-defined structures.

Compouds	Year	Ref.
$\{O[Ru^{IV}(X)P_2W_{17}O_{61}]_2\}^{16-}$ (X = OH, Cl)	1993	S1
$\{[(WZnRu_2^{III}(OH)(H_2O)](ZnW_9O_{34})_2\}^{11-}$	1995	S2
$\{[(Zn_2Ru_2^{III}(OH)(H_2O)](ZnW9O_{34})_2\}^{14-}$	2004	S3
$[SiW_{11}O_{39}Ru^{III}(H_2O)]^{5-}$	2006	S4
$[\{a\text{-}SiW_{11}O_{39}Ru^{III}\}_2O]^{12-}$	2007	S5
$[\{PW_{11}O_{39}\}_2\{(HO)Ru^{IV}\text{-O-}Ru^{IV}(OH)\}]^{10-}$	2008	S6
$[\{Ru^{IV}_4O_4(OH)_2(H_2O)_4\}(\gamma\text{-}SiW_{10}O_{36})_2]^{10-}$	2008	S7
$[Ru^{IV}_4Cl_4O_2(\mu\text{-OH})_4(\gamma\text{-}SiW_{10}O_{36})_2]^{12-}$	2008	S8
$[\gamma\text{-}XW_{10}O_{38}\{Ru^{IV}N\}_2]^{6-}$ (X = Si, Ge)	2009	S9
$[(\gamma\text{-}PW_{10}O_{36})_2Ru^{IV}_4O_5(OH)(H_2O)_4]^{9-}$	2010	S10
$[\{Ru^{IV}_4O_6(H_2O)_9\}_2Sb_2W_{20}O_{68}(OH)_2]^{4-}$	2012	S11
$[\{Ru^{IV}_4O_6(H_2O)_9\}_2\{Fe(H_2O)_2\}_2\{\beta\text{-}TeW_9O_{33}\}_2H]^{4-}$	2012	S11

Table S2. The bond valence sum calculations of all crystallographically unique molybdate and ruthenium atoms on **1a**.

Atom lable	BVS	Atom lable	BVS
Mo1	6.03	Mo5	5.99
Mo2	6.02	Mo6	6.10
Mo3	6.03	Mo7	6.02
Mo4	6.09	Ru1	3.97

Table S3. The bond valence sum calculations of all crystallographically unique oxygen atoms on **1a**.

Atom	BVS	Atom	BVS
O1	1.69	O14	1.77
O2	1.71	O15	1.81
O3	1.98	O16	1.72
O4	1.76	O17	1.73
O5	1.19	O18	1.73
O6	1.91	O19	1.73
O7	1.63	O20	2.07
O8	1.85	O21	2.08
O9	1.84	O22	1.69
O10	1.66	O23	1.88
O11	1.79	O24	1.74
O12	1.82	O25	1.91
O13	1.65	O26	1.89

Table S4. Optimization of catalytic oxidation of 1-phenylethanol

Entry	Catalyst (mol%)	Temp. (°C)	TBHP (equiv.)	Time (h)	Yield[a] (%)
1	0.15	85	8.0	3	100
2	0.10	85	8.0	3	93
3	0.05	85	8.0	3	86
4	0.03	85	8.0	3	84
5	0.15	85	7.0	3	95
6	0.15	85	6.0	3	93
7	0.15	85	5.0	3	62
8	0.15	75	8.0	3	97
9	0.15	60	8.0	3	75
10	0.15	50	8.0	3	46
11	0.15	85	8.0	2	94
12	0.15	85	8.0	1	83
13	0.15	85	8.0	0.5	54
14[b]	–	85	8.0	3	27
15[c]	0.30	85	8.0	3	80
16[d]	2.10	85	8.0	3	33

Reaction conditions for the entries 1 to 16: substrate (1 mmol) and acetonitrile (3 mL). [a]Yield determined by GC with dodecane as an internal standard. [b]Blank experiment: the reaction was carried out without catalyst **1**. [c]$RuCl_3{\cdot}nH_2O$ used as a catalyst. [d]$Na_2MoO_4{\cdot}2H_2O$ used as a catalyst.

Table S5. Crystallographic data of **1**.

	**1**
Empirical formula	$Cs_3H_{48}Na_6O_{76}Mo_{14}Ru_2$
Formula weight	3346.35
Crystal system	triclinic
Space group	*P*-1
a [Å]	9.846(7)
b [Å]	14.133(10)
c [Å]	14.335(10)
α /°	118.558(14)
β /°	97.674(14)
γ /°	95.575(16)
Volume/$Å^3$	1706(2)
Z	2
ρcalcg/cm^3	3.256
μ/mm^{-1}	4.654
F(000)	1563.0
Crystal size/mm^3	0.37 × 0.22 × 0.18
Data/parameters	6013/275
R_{int}	0.0156
Goodness-of-fit on F^2	1.023
*R*1, *wR*2 [$I \geq 2\sigma$ (I)]	0.0435, 0.110
*R*1, *wR*2 [all data]	0.0509, 0.117

References:

S1. Randall, W.J.; Weakley, T.J.R.; Finke, R.G. Oxidation Resistant Inorganic-Porphyrin Analog Polyoxometalates. 3. The Synthesis and X-ray Crystallographic Characterization of a New Heteropolyoxoanion Structural Type, the Diruthenium-Oxo-Bridged " Rimetallic Inorganic-Porphyrin Analog" $KLi_5[O\{RuCI(\alpha_2\text{-}P_2W_{17}O_{61})\}_2]\cdot 2KC1\cdot 60H_2O$. *Inorg. Chem.* **1993**, *32*, 1068–1071.

S2. Neumann, R.; Khenkin, A.M. Noble Metal (Ru^{III}, Pd^{II}, Pt^{II}) Substituted "Sandwich" Type Polyoxometalates: Preparation, Characterization, and Catalytic Activity in Oxidations of Alkanes and Alkenes by Peroxides. *Inorg. Chem.* **1995**, *34*, 5753–5760.

S3. Howells, A.R.; Sankarraj, A.; Shannon, C. Diruthenium-Substituted Polyoxometalate as an Electrocatalyst for Oxygen Generation. *J. Am. Chem. Soc.* **2004**, *126*, 12258–12259.

S4. Sadakane, M.; Tsukuma, D.; Dickman, M.H.; Bassil, B.; Kortz, U.; Higashijima, M.; Ueda, W. Structural characterization of mono-ruthenium substituted Keggin-type silicotungstates. *Dalton Trans*. **2006**, 4271–4276.

S5. Sadakane, M.; Tsukuma, D.; Dickman, M.H.; Bassil, B.S.; Kortz, U.; Capron, M.; Ueda, W. Dimerization of mono-ruthenium substituted *a*-Keggin-type tungstosilicate [*a*-

$SiW_{11}O_{39}Ru^{III}(H_2O)]^{5-}$ to μ-oxo-bridged dimer in aqueous solution: synthesis, structure, and redox studies. *Dalton Trans.* **2007**, 2833–2838.

S6. Chen, S.-W.; Villanneau, R.; Li, Y.; Chamoreau, L.-M.; Boubekeur, K.; Thouvenot, R.; Gouzerh, P.; Proust, A. Hydrothermal Synthesis and Structural Characterization of the High-Valent Ruthenium-Containing Polyoxoanion $[\{PW_{11}O_{39}\}_2\{(HO)Ru^{IV}–O–Ru^{IV}(OH)\}]^{10–}$. *Eur. J. Inorg. Chem.* **2008**, 2137–2142.

S7. (a) Sartorel, A.; Carraro, M.; Scorrano, G.; Zorzi, R.D.; Geremia, S.; McDaniel, N.D.; Bernhard, S.; Bonchio, M. Polyoxometalate Embedding of a Tetraruthenium(IV)-oxo-core by Template-Directed Metalation of $[\gamma\text{-}SiW_{10}O_{36}]^{8–}$: A Totally Inorganic Oxygen-Evolving Catalyst. *J. Am. Chem. Soc.* **2008**, *130*, 5006–5007; (b) Geletii, Y.; Botar, B.; Kögerler, P.; Hillesheim, D.; Musaev, D.; Hill, C.L. An all-inorganic, stable, and highly active tetraruthenium homogeneous catalyst for water oxidation. *Angew. Chem. Int. Ed.* **2008**, 47, 3896–3899.

S8. Yamaguchi, S.; Uehara, K.; Kamata, K.; Yamaguchi, K.; Mizuno, N. A γ-Keggin-type dimeric silicotungstate sandwiching an adamantanoid tetra-nuclear ruthenium–oxygen cluster core. *Chem. Lett.* **2008**, *37*, 328–329.

S9. Besson, C.; Musaev, D.G.; Lahootun, V.; Cao, R.; Chamoreau, L.-M.; Villanneau, R.; Villain, F.; Thouvenot, R.; Geletii, Y.V.; Hill, C.L.; Proust, A.; Vicinal Dinitridoruthenium-Substituted Polyoxometalates $\gamma\text{-}[XW_{10}O_{38}\{RuN\}_2]^{6–}$ (X = Si or Ge). *Chem. - Eur. J.* **2009**, *15*, 10233–10243.

S10. Besson, C.; Huang, Z.; Geletii, Y.V.; Lense, S.; Hardcastle, K.I.; Musaev, D.G.; Lian, T.; Proust, A.; Hill,C. L. $Cs_9[(\gamma\text{-}PW_{10}O_{36})_2Ru_4O_5(OH)(H_2O)_4]$, a new all-inorganic, soluble catalyst for the efficient visible-light-driven oxidation of water. *Chem. Commun.* **2010**, *46*, 2784–2786.

S11. Kalinina, I.V.; Izarova, N.V.; Kortz, U. Bis[tetraruthenium(IV)]-Containing Polyoxometalates: $[\{Ru^{IV}{}_4O_6(H_2O)_9\}_2Sb_2W_{20}O_{68}(OH)_2]^{4–}$ and $[\{Ru^{IV}{}_4O_6(H_2O)_9\}_2\{Fe(H_2O)_2\}_2\{\beta\text{-}TeW_9O_{33}\}_2H]^{-}$. *Inorg. Chem.* **2012**, *51*, 7442–7444.
